# Supplementary material for: Are carbon emissions trading and green financial instruments synergistic? -Comprehensive quantitative research based on content analysis
Source: PLoS One. 2024 Mar 7;19(3):e0298601. doi: 10.1371/journal.pone.0298601 (PMC10919601; doi:10.1371/journal.pone.0298601)
Supplement: S1 Data — (ZIP) [file pone.0298601.s001.zip › Data/地级市Z_F工作报告.zip!/╔╜╬≈/╦╖╓▌╩╨/└┤╫╘╣1⁄2╓┌║┼í╛┬φ┐╦╩2╛▌═°í┐_2013.docx]

各位代表：

　　现在，我代表市人民政府向大会作工作报告，请予审议，并请市政协委员和其他列席人员提出意见。

　　一、2012年工作回顾

　　2012年，我们认真贯彻落实党的十八大精神和省委、省政府的决策部署，大力弘扬右玉精神，坚持主题主线，加快推进工业新型化、农业现代化、市域城镇化、城乡生态化，紧紧围绕“坚定不移推进转型跨越发展，全力打造新基地新优势新朔州”的工作主题和“建设自然、生态、现代、宜居幸福新城”的宏伟目标，牢牢把握稳中求进的工作总基调，坚持发展不松劲，狠抓转型不动摇，精心谋划新举措，全力以赴抓落实，全市经济社会发展保持平稳较快、健康协调的良好态势。

　　(一)综合经济实力跃上新台阶。坚持把稳增长作为首要任务，及时研究出台了加强项目建设、扶持服务业发展、促进小微企业健康发展等一系列措施。全市实施投资1000万元以上的项目373个，完成投资977亿元，其中，省市重点工程258项，完成投资917.2亿元。市财政投入1亿多元，扶持重点企业和项目。在项目建设拉动、实体经济支撑、政策措施保障等多方面积极因素的作用下，全市经济保持全省领先的发展势头。全市地区生产总值达到1007.1亿元，增长11.1%;工业增加值完成569.9亿元，增长16.3%;服务业增加值完成360.14亿元，增长9.5%;全社会固定资产投资完成610.6亿元，增长28.6%;财政总收入完成210亿元，增长20%;一般预算收入完成84.25亿元，增长18.8%;社会消费品零售总额达到191.1亿元，增长16.5%;外贸进出口总额达到2.57亿美元，增长84.2%;城镇居民人均可支配收入达到23114元，增长13.9%;农民人均纯收入达到8000元，增长13.9%。主要经济指标均超额完成目标任务，10项主要经济指标有8项增幅位居全省前三。

　　(二)安全生产水平得到新提升。始终坚持把安全生产工作放在突出位置，常抓不懈。认真落实政府安全监管和企业安全生产两个主体责任，扎实开展安全生产年活动。市政府在安全监管方面投入2163万元，建设了安全监管综合信息平台，组建了17支专家队伍，配齐配强了基层安监机构。市政府领导实行联系县区、联系煤矿制度，经常深入企业调研指导安全生产工作。有关部门对近几年发生的典型事故进行了剖析、通报，深入开展了以煤矿为重点的各行业各领域安全生产专项整治和“打非治违”专项行动。各重点行业全面推行企业安全生产承诺制，积极推进企业安全生产标准化建设，安全管理得到加强，安全生产保障水平得到提高。全年事故起数和死亡人数分别比上年下降14.94%和26.74%，安全生产形势进一步好转。

　　同时，全面加强社会管理综合治理，健全社会治安防控体系，认真做好信访工作，妥善处理各类社会矛盾，保持了社会和谐稳定，为党的十八大胜利召开发挥了“护城河”作用。

　　(三)产业转型升级迈出新步伐。坚持把调结构、促转型作为转变经济发展方式的主攻方向，不断加大工作力度。加快推进工业新型化。积极推进现代化矿井建设，兼并重组保留矿井全年完成改造投资100多亿元，22座矿井达到标准化矿井要求，全市原煤产量达到2.07亿吨。积极发展新能源电力，电力装机容量达到633.85万千瓦;其中，煤矸石发电装机容量达到267万千瓦，居全国第一;风电装机容量达到81.75万千瓦，居全省第一。加快提升陶瓷产业发展水平，全市日用瓷生产能力达到16亿件。特别是狠抓了八个工业园区和经济开发区，新兴产业和循环经济加快发展。平朔大厢斗、应县汽车产业园、右玉永昌科技半导体照明及芯片制造等89项新兴产业重点项目，完成投资70多亿元，新兴产业投资增幅达到69%。中源伟业粉煤灰制备陶瓷纤维等项目建成投产，中煤平朔粉煤灰提取氧化铝白炭黑、劣质煤综合利用等循环经济高端项目积极推进，具有朔州特色的循环经济取得新突破。八个工业园区投产和在建项目达到314个，总资产达到520多亿元，2012年实现产值256亿元，比上年增长70%多，成为全市重要的经济增长极。园区管理机构组建运行，园区建设走上正轨。加快提升农业现代化。大力推进“一村一品、一县一业”，特色农业加快发展。粮食产量达到21.4亿斤，增长10.2%，创历史新高。新增设施农业面积3.15万亩，总面积达到10万亩。新建和完善标准化养殖园区116个，奶牛养殖园区达到236个，肉羊养殖园区达到187个，奶牛存栏达到17.8万头，肉羊饲养量达到403万只。怀仁羔羊肉、右玉羔羊肉获得国家原产地地理标识保护认证，应州绿蔬菜种植基地通过中国质量认证中心整体验收。积极扶持农业产业化龙头企业，农产品品牌建设得到加强。农产品加工龙头企业达到202个，18家企业年销售收入超亿元，古城集团销售收入达到8亿元，全市农产品加工企业销售收入超过100亿元。在第二届山西特色农产品北京展销周上，签约招商引资项目35个，引资额243亿元;签约贸易项目74个，总金额达到64.5亿元;现场销售各类优质农产品达1118万元;特别是推出的朔州十大优质农产品深受北京市民喜爱，供不应求，鑫邦燕麦还成功打入韩国市场。加速发展服务业。怀仁海宁皮革城、朔城区义乌小商品城、戴斯大酒店等项目建成运营。文化旅游业加快发展，2012年旅游业收入达到61.6亿元，增长35.5%。强化科技人才对转型的支撑作用。实施“三个一百”科技人才引进计划，聘请各方面专家100多名，联系高校47所，招录100多名研究生到市直部门工作，引进北京大学2名博士挂职，吸引20名博士来朔工作。加大科技研发支持力度，全市财政安排科技研发经费6709万元，企业研发中心达到23家，全市申报专利578件。市政府投资3000万元，与北京大学合作建成固废资源化研究中心。

　　(四)城乡环境面貌呈现新变化。进一步加大城镇化推进力度，全市铺开城镇化项目246项，全年完成投资154亿元。加快建设中心城市。继续推进道路、水源、供热、绿化、净化、亮化六大市政工程，改造20多条城市道路，增加集中供热面积600万平方米，城市功能进一步完善。深入推进特色城镇建设。各县城加快扩容提质，五个省级百镇建设示范镇启动建设。2012年底，全市城镇化率达到50.02%，城镇化水平实现历史性突破。进一步推进新农村建设。增加重点推进村185个，总数达到818个。深入开展干部包村增收和机关定点扶贫活动，1.3万贫困人口实现脱贫。普遍开展环境综合治理，农村面貌得到改变。大力加强交通建设。全市公路建设完成投资97亿元，公路通车里程达到9910公里。加大造林绿化力度。广泛种植大规格苗木，全市投资15.2亿元，高标准完成营造林33.2万亩。大力加强水系建设。投资19.8亿元，建设33处水系重点工程。狠抓节能减排，强力淘汰落后产能。市区取缔了11台供热锅炉，关停了全部污染企业。

　　(五)民生工作取得新成果。财政支出进一步向民生倾斜，全市财政在民生领域投入89.3亿元，占一般预算支出的64.36%。加大教育投入力度。新增39所城镇和农村幼儿园。实施义务教育阶段学校标准化建设，“两基”工作受到国务院表彰。加强教学管理，高中教育水平进一步提高。倾力发展高等教育。自筹资金6亿元，建成了我市第一所本科院校中北大学朔州电力学院。经过积极努力，朔州师专获得省政府批准。推动文化体育事业发展。应县木塔被列入世界文化遗产预备名录。在山西省第十四届运动会阶段赛上，我市运动员夺得6金7银5铜的好成绩，竞技体育取得进步。深化医疗卫生体制改革。基本药物制度由乡村卫生服务机构扩大到试点县县级公立医院。实施积极就业政策。新增就业人数2.2万人，完成省定目标任务的147%。进一步健全社会保障体系。启动五险统征工作，提高城乡低保和居民医保标准，发放社会保障卡100万张。积极救助困难群众。与此同时，加强国防后备力量建设，积极发展人口计生、妇女儿童、老龄和残疾人事业，民族、宗教、人防、地震、应急、气象、档案、地方志等各方面工作都取得新成绩。

　　特别是大力实施惠民工程。完成778个农村5946公里街巷硬化，建成378个便民连锁店，圆满完成农村新的“五个全覆盖”工程。开工建设各类保障性住房41431套，完成投资60.69亿元。改造农村危房9248户，完成投资2.4亿元。为40.54万农户免费供应了冬季取暖煤。市政府投入1000多万元，购置20部新型环保公交车投入运营。投入8000多万元，解决了遗留多年的7家改制企业1100多名职工的安置问题。投入9000万元，实施义务教育阶段寄宿学生饮用奶工程，免除城市义务教育阶段学生教科书费用，开展送戏下乡活动，实施爱心助残工程，建设市社会福利院、儿童福利院，建成商贸创业孵化基地和市劳动力市场，为市区残疾人、伤残军人和70岁以上老年人发放公共交通补贴，市政府承诺为民兴办的八件实事全部落实到位。

　　(六)改革开放实现新突破。全面展开转型综改试验工作。市级和平鲁区、右玉县转型综改行动方案都获得批复。“一市两县”、“一市两园”、“一县一企”和转型标杆项目扎实推进。土地保障机制得到创新。争取到国家和省批复多项试点政策，保障了工业化和城镇化建设用地。金融体系加快完善。我市被列为全省资本市场支持转型综改试点市。晋商银行、浦发银行朔州分行正式开业，全市银行业机构达到14家。各家金融机构强化服务意识，加大信贷投放力度，当年新增存贷比达到80%，有力地支持了朔州发展。产业转型促进机制加快建立。坚持以工补农，推进“一矿一企”， 煤炭企业在循环经济、装备制造、现代农业、现代服务业等领域兴办了大批转型项目。我市广大企业家为转型跨越发展做出了积极贡献。

　　加大招商引资力度。精心组织参加中部博览会、能源博览会、晋商大会、广州招商会等大型招商活动，赴台湾开展旅游推介活动，成功举办了首届山西省工艺美术精品博览会暨朔州精品陶瓷展、第一届中国朔州煤炭工业及循环利用技术装备展览会。2012年，签约招商引资项目178项，总投资3704亿元，签约项目总投资额居全省第三;到位外来投资684亿元，到位额居全省第一。

　　(七)政府自身建设取得新进步。自觉接受人大和政协监督，积极支持各民主党派、工商联、人民团体和无党派人士参政议政，全年共办理人大代表建议和政协委员提案247件。积极推进行政审批制度改革和政务公开，市政府各部门减少了193项行政审批事项，保留的143项审批许可事项全部按照“两集中”、“两到位”的要求进入政务服务中心集中办理。着力提高行政效能，开通了项目审批绿色通道，建立了公共资源交易平台。着力转变工作作风，市政府各单位积极开展“六个一”活动。加强法制建设，“六五”普法深入开展。加强廉政建设，强化行政监察和审计监督，深入开展煤焦、工程建设等重点领域专项治理，“一网六平台”惩防体系信息网建设全省领先。

　　各位代表，2012年是我市经济社会发展跃上新台阶、实现新跨越的一年，中央领导和省委、省政府对朔州工作给予殷切关怀和高度评价。9月28日，习近平总书记再次就学习弘扬右玉精神作出重要批示，充分肯定右玉精神的可贵之处，对学习弘扬右玉精神、深入贯彻落实科学发展观、牢固树立正确政绩观提出明确要求。8月份，国务院在我市召开三北防护林工作会议，国务院领导肯定我们的造林绿化工作“可圈可点、可喜可贺、可歌可泣、可看可学”。9月份，在全省观摩检查活动中，省委袁纯清书记肯定我市“发展局面蓬勃向上，发展势头欣欣向荣，发展成果令人振奋”。在全省2012年度目标责任考核中，我市被省委、省政府表彰为优秀市。我市还被表彰为全省安全生产工作模范市、造林绿化先进市、农民增收先进市、社会管理综合治理工作先进市，被授予省级环保模范城市、省级创业型城市称号，被有关部门评为影响中国改革的十大资源型城市。

　　各位代表，过去的一年，我们在宏观经济形势比较困难的情况下取得这些成绩，实属不易。这是省委、省政府和市委正确领导的结果，是市人大、市政协有力监督和鼎力支持的结果，是全市人民齐心协力、共同奋斗的结果。在这里，我代表市人民政府，向全市人民和所有关心支持朔州发展的各界人士，表示崇高的敬意和衷心的感谢!

　　在肯定成绩的同时，我们也要清醒地看到，我市经济社会发展中不平衡、不协调、不可持续的问题还比较突出。新兴产业占比小，支柱产业仍然单一，经济结构不优;安全生产和节能降耗压力较大，转变经济发展方式任务艰巨;农业基础薄弱，农民持续增收难度较大;社会事业发展滞后，城市功能还不完善，城镇化质量不高，推进城乡发展一体化任务艰巨;政府职能和干部作风需要进一步转变，发展环境需要进一步优化，等等。对此，我们一定要高度重视，认真加以解决。

　　二、2013年工作安排

　　2013年是全面贯彻落实党的十八大精神的开局之年，是实施“十二五”规划承前启后的关键一年，是为全面建成小康社会奠定坚实基础的重要一年。

　　新的一年，朔州发展站在了新的起点上。从宏观经济形势看，我国经济社会发展长期向好的基本趋势没有变。但是，我国经济增长下行压力有所加剧，煤炭市场持续低位运行。我市经济连续多年高位运行，基数不断扩大，保持经济持续较快增长的难度越来越大。我们既要看到形势的积极方面，坚定信心，也要看到形势的复杂性，增强忧患意识和紧迫感，兢兢业业、扎扎实实做好工作。

　　今年工作的总体要求是：全面贯彻落实党的十八大精神，高举中国特色社会主义伟大旗帜，以邓小平理论、“三个代表”重要思想、科学发展观为指导，深入贯彻落实习近平总书记学习弘扬右玉精神的重要批示精神，按照“安全稳定促和谐，抢抓机遇树标杆，整体工作上台阶”的要求，紧紧围绕优化经济结构和提升发展质量两大任务，以转型综改试验区建设为统领，深化改革开放，强化创新驱动，加快推进工业新型化、农业现代化、市域城镇化和城乡生态化，全面推进经济、政治、文化、社会和生态文明建设，着力夯实安全生产基础，着力保持经济持续健康发展，着力发展循环经济，着力推进创新驱动，着力保障和改善民生，努力建设国家重要的煤电基地、综合能源基地和循环经济示范基地，建设生态畜牧基地和特色农产品基地，建设边塞文化旅游目的地和北方避暑胜地，率先走出资源型地区转型跨越发展的新路子。

　　今年经济社会发展的主要预期目标是：地区生产总值完成1130亿元，增长11%;工业增加值完成650亿元，增长14%;服务业增加值完成400亿元，增长9%;固定资产投资完成757.2亿元，增长24%;财政总收入完成239.4亿元，增长14%;公共财政预算收入完成95.2亿元，增长13%;社会消费品零售总额完成220亿元，增长15%;城镇居民人均可支配收入达到25660元，增长11%;农民人均纯收入达到8960元，增长12%;城镇新增就业岗位2.2万个，城镇登记失业率控制在4.2%以内;居民消费价格涨幅控制在3.5%左右。

　　约束性指标是：万元地区生产总值综合能耗下降3.6%，二氧化硫、化学需氧量、氨氮、氮氧化物减排完成省下达任务，烟尘、粉尘排放量均下降2%，万元工业增加值用水量下降5.2%。

　　主要工作任务是：

　　(一)牢牢抓住扩大内需机遇，促进经济持续健康发展

　　加大项目建设力度，发挥好投资对经济增长的关键作用。深入开展“项目推进年”活动。实行项目储备、签约、落地、开工、建设和投产“六位一体”工作机制，进一步加大重点工程建设力度，扩大投资规模。全市计划实施1000万元以上的项目631项，当年计划完成投资975.6亿元，其中，省市重点建设项目178项，年度计划投资784亿元。今年的投资规模为历年之最。在投资结构上，着力加强重大基础设施、产业开发、民生改善、生态建设等重点领域。在资金来源上，通过政府投资、银行融资、企业自筹和招商引资等多种渠道筹集资金。积极支持中煤平朔实施“双千亿”工程，中煤金海洋实施“双五百亿”工程，中电国际、同煤集团、山西煤销集团实施“双百亿”工程，充分发挥大企业的主力作用。政府要创造良好环境，鼓励引导更多的民间资本投资本地，建设家乡。金融机构要更积极地支持地方建设，把更多的信贷资金投放到当地，发挥好对投资的促进作用。

　　加大对企业的支持力度，发挥好实体经济对经济增长的支撑作用。实行更加有利于实体经济发展的财政、金融等政策措施，引导更多的资本、技术、人才等要素投向实体经济。加强对各类企业的支持和服务。加大对中小企业产品开发、品牌创建、市场开拓的支持力度，培育更多销售收入超亿元的小巨人企业，使更多的企业进入全省百强。落实支持小微企业发展的政策措施，加强融资担保、创业辅导、企业孵化、人才培训等服务，为小微企业发展创造有利条件。

　　加快发展服务业，增强消费对经济增长的拉动作用。落实促进消费的政策措施，加快培育一批拉动力强的新的消费增长点，提升消费环境，让更多的消费留在本地。突出抓好服务业项目。积极推进平朔物流园区、应县天津港综合物流园区等项目，进一步做强煤炭物流业。加快建设朔州老城、朔州数码港、怀仁海宁皮革城二期、怀仁商业步行街等项目，积极推进新时代广场、清河湾等城市综合体，着力打造新型商务区和特色商业街，进一步提升商贸流通业水平。继续推进应县木塔申遗工作，加大崇福寺等文化旅游资源宣传开发力度，提升旅游业发展水平。鼓励汽车、家电、住房装修等大宗消费，促进家政、养老、文化、娱乐等服务消费，活跃消费市场。

　　(二)深入推进工业新型化，着力构建现代产业新体系

　　大力发展循环经济。把循环经济作为朔州发展的一条基本路径，按照路径最多元、产品最多样、技术水平最高端“三个最”的要求，以建设全国工业固废综合利用示范基地为平台，以多联产、全循环、高端化为基本模式，改造提升传统产业，延伸壮大煤基产业链，促进黑色煤炭绿色发展，高碳产业低碳发展。

　　巩固提升煤炭资源整合煤矿兼并重组成果，加快建设规模化、集约化、机械化、信息化的现代化矿井，年内力争建成30座标准化矿井。加快发展电力工业，支持煤矸石发电、风电、太阳能发电、生物质能发电等新能源电力项目。推动神头发电公司2×60万千瓦机组和平鲁大山台20万千瓦风电等项目早日投产，推动神头发电公司2×100万千瓦、中煤平朔2×66万千瓦煤矸石发电示范项目、开发区华电2×30万千瓦热电联供项目加快建设。这些项目建成运行后，我市将成为千万千瓦级电力基地。鼓励煤电企业联营，构建和谐煤电关系。适应市场变化，做好煤炭产运销衔接工作。突出抓好循环经济高端项目，推动中源伟业粉煤灰制备陶瓷纤维等项目达产达效，推动平朔粉煤灰提取氧化铝白炭黑项目、劣质煤综合利用等项目加快建设，推动平朔煤矸石电厂超细粉煤灰等项目开工建设。

　　加快发展新兴产业。要把加快发展新兴产业作为转型发展的着力点，不断加大工作力度。进一步做大做强陶瓷、特色农畜产品加工等具有一定基础和比较优势的产业，大力发展装备制造、劣质煤综合利用等市场前景好的产业，积极发展节能环保、信息技术等成长性好的高新技术产业，加快形成支柱产业多元化的格局。每个县区都要建设一批投资在5亿元以上的循环经济、新兴产业项目。着力抓好开发区安普电动汽车、朔城区电动汽车、应县北方汽车产业园、右玉永昌科技半导体照明及芯片制造、怀仁玉龙集团精细化工产业园、古城乳业新园区、山阴鑫邦燕麦新生产线等项目，建设好一批新兴产业龙头项目，发挥好示范带动作用。

　　实施创新驱动发展战略。科技创新是提高企业核心竞争力的关键，必须把科技创新摆在更加突出的位置。推动科技与经济紧密结合，着力构建以企业为主体、市场为导向、产学研相结合的技术创新体系。企业要发挥好主体作用，加大科技研发投入力度。工业园区、重点企业都要加强与高等院校、科研机构的联系合作，引进高层次人才，建立技术研发中心，提高自主创新能力。巩固去年科技人才“三个一百”活动成果，积极推进与北京大学、清华大学等高校的合作，推动固废综合利用、新能源汽车等项目取得实效。政府要加大科技研发投入力度,积极扶持重大科技攻关项目，着力培育一批科技型中小企业。鼓励企业运用信息技术改造工艺流程，推动信息化与工业化深度融合。

　　着力抓好经济开发区和八个工业园区。经济开发区和八个工业园区要按照空间集中开发、资源集约利用、产业集群发展、服务集聚配套的要求，创新管理机制，完善发展规划，加强基础设施建设，以发展循环经济和新兴产业为重点，大上项目，上大项目，上好项目，加快提高发展水平和经济实力，真正成为朔州转型跨越发展的重要载体。开发区要努力创建国家级开发区，八个工业园区要争创省级开发区。固废综合利用工业园区，要加强与北京大学的合作，打造全国粉煤灰综合利用示范基地。朔城区富甲工业园区，要以装备制造、新材料为主导产业，建设新兴产业集聚区。东露天循环经济园区，要推进煤电一体化，提高资源就地转化率。平鲁北坪工业园区、山阴北周庄工业园区，要立足多联产、全循环，打造煤基循环经济工业园区。怀仁金沙滩陶瓷工业园区，要注重产业整合，加快提档升级。应县新型产业科技园区，要积极引进高新技术，发展新能源、高端陶瓷和文化创意等产业。右玉梁威工业园区，要突出生态绿色，着力发展特色农产品深加工产业。

　　(三)深入推进农业现代化，当好雁门关生态畜牧经济区建设排头兵

　　进一步加大强农惠农富农政策力度。在落实好中央和省各项强农惠农富农政策的基础上，我市要在“三农”领域投入更多资金，加大农业生产补贴力度，加强农业农村基础设施建设，推动资源要素向农村配置，让公共财政的阳光更多地照耀农村，真正让农民种粮务农有效益、不吃亏、得实惠。继续实施百万亩玉米地膜覆盖等富民工程，实行规模种植、规模养殖政策性农业保险财政补贴，让广大农民得到更多实惠。

　　大力发展特色现代农业。我市特色农业优势明显，一定要做好特色农业这篇大文章。继续推进“一村一品、一县一业”，新增100个省级专业村。大力推进现代农业示范园区建设，今年新增设施农业4万亩。进一步推进规模健康养殖，新建和提升标准化养殖园区50个，发展10个千头现代化奶牛场，建设9万亩优质人工草场，奶牛存栏达到18万头，肉羊饲养量达到430万只。积极引进国外生物活性水技术，加快提高农业循环经济发展水平。农业循环经济要实现“三个全”，玉米和秸秆全部被畜牧业利用，农畜产品全部深加工，牲畜粪便全部生物化处理、资源化利用，生态畜牧基地发展水平显著提高，走出以畜牧养殖和农畜产品加工为重点的农业现代化路子。

　　加快提高农业产业化发展水平。扶持古城、天鹏、雅士利、右玉臣丰、怀仁五洲等农产品加工龙头企业做大做强，着力打造一批销售收入上亿元、5亿元、10亿元的龙头企业集群。今年农产品加工企业销售收入要达到120亿元。鼓励农产品加工企业整合重组，培育壮大龙头企业。怀仁、右玉要整合羊肉加工企业，积极发展基础母羊，带动养羊产业持续健康发展。大力提升优质农产品精深加工水平，推动十大优质农产品在全省全国叫响品牌。加强农田水利基本建设，提高农业综合生产能力。推动农业科技创新，加快农业机械化进程。完善农业科技推广服务体系，逐步充实乡镇畜牧兽医队伍。创新农业生产经营体制机制，推广公司加基地、基地连农户的产业化经营模式，提高农业集约化、专业化、组织化、社会化程度。

　　扎实推进新农村建设。结合农村危房改造、矿区塌陷区治理、移民搬迁、饮水解困、土地整治，进一步推进新农村建设。新增120个重点村。加大农村环境整治力度，不断改善农村面貌。在巩固提升过去两轮“五个全覆盖”成果的基础上，未来五年，为农民再办五件实事，即全面完成农村困难家庭危房改造、特困群众易地搬迁、行政村街道亮化、村级幼儿园改扩建和乡村清洁工程。加大扶贫开发力度，深入开展干部下乡住村、领导干部包村增收活动和机关单位定点扶贫工作，今年完成整村推进20个村，易地搬迁贫困人口2238人，实现1.6万人稳定脱贫。

　　(四)加快推进城镇化进程，推动城乡发展一体化

　　继续抓好中心城市建设。深入推进国家园林城市、国家卫生城市创建活动，加快建设自然生态现代宜居幸福新城。进一步推进道路、水源、供热、绿化、净化、亮化等市政工程建设，完善公共服务条件，提高居民生活便利度和舒适度。道路建设方面，抓好恢河大桥、平朔南路、和平街续建，改扩建马邑北路、北关路，建设环城高速西出口、南出口、东南出口。绿化方面，重点搞好环城高速西出口、南出口、东南出口以及建设南路等五条道路绿化。供水方面，建设四水厂、市污水处理厂中水回用工程、第二污水处理厂。供热方面，建设热电联供三期工程，新增供热面积600万平方米。公共服务设施方面，规划建设大医院、科技馆、体育中心等项目。坚持因地制宜，加快推进城中村改造，改善居住条件，改变城市面貌。坚持公交优先发展，突出城市公共交通的公益性，加快完善城市公共交通运营服务体系，为广大群众出行提供便捷服务。理顺城市规划建设管理体制，加强城市管理，启动数字城管工程，着力整治交通秩序、环境卫生、市场管理等方面的突出问题，提高城市整洁度，促进城市协调有序发展。

　　积极推进以县城为重点的城镇建设。继续实施大县城战略，打造各具特色的工业强镇、商贸重镇和旅游名镇。平鲁区要以工业化为引领，按照工业化、信息化、城镇化、生态化、农业现代化五化联动的思路，大力实施“一城四镇三十个新型农村社区”规划，统筹推进户籍、住房、就业和社会保障等工作，建设“智慧平鲁”，力争早日实现“70%的土地种草种树、70%的农民进城”。右玉县要以生态为基，坚持生态优先，在巩固提升生态建设成果的基础上，大力发展生态农业、生态工业、生态城镇和生态文化旅游产业，做好生态文章。怀仁县要以服务业带动城市发展，以商贸物流业带动经济发展，打造华北最美县城。山阴县要大力发展循环经济，依托桑干河流域治理，建设生态新城。应县要依托木塔申遗工作，积极打造旅游文化名城。实施以五个省级示范镇为重点的十镇建设工程，统筹各项政策措施，完善公共服务设施，促进产业和人口集聚，形成城镇集群发展的格局。

　　加快交通网络建设。计划实施4条高速公路、4条干线公路、4条重点公路和5条旅游公路，建成460公里，完成投资22亿元。积极推进西纵高速朔州段、环城高速东北段等公路建设，年内实现环城高速西南段、荣乌高速山阴至平鲁段和生态观光旅游公路建成通车。加强公路养护管理，保障公路安全畅通。

　　统筹城乡发展。城镇化是现代化建设的历史任务，城乡发展一体化是解决“三农”问题的根本途径。全面推进农村改革试验区工作，积极探索城乡发展一体化新途径。坚持中心城市、大县城、小集镇、新农村、工业园区统筹发展战略，推进城乡规划、基础设施、产业发展、生态环境、公共服务“五个一体化”，促进城乡之间有机融合、联动发展。在教育、就业、社会保障等方面，统筹推进城乡基本公共服务均等化。研究出台户籍制度改革实施意见，有序推进农业转移人口市民化。

　　(五)牢固树立生态文明理念，努力建设美丽朔州

　　继续大力推进造林绿化。抓好山上治本。大力推进连片荒山造林工程，加快增加森林资源总量，争取森林覆盖率每年提高一个百分点。抓好身边增绿。每个县区都要精心打造几个万亩以上的城郊森林公园和生态景区。城郊森林公园要按照“市场化运作、公司化承载、园区化打造”的模式，鼓励社会资本参与建设。按照“一乡一条路，一村一片林，一人一棵树”的要求，每个乡镇都要高标准绿化一条主干道路，每个村庄都要种植一片环村林，每家每户都要因地制宜在房前屋后栽植树木，绿化庭院。抓好生态富民。坚持“生态建设产业化，产业发展生态化”的理念，积极发展经济林和林下经济，着力建设大型苗圃，增强林业自身发展能力，带动农民增收致富。今年要完成33万亩的营造林任务，重点抓好“四个六”工程，即6条通道绿化、6大生态景区、6大规模造林工程和6大苗木基地。同时，加强森林资源管护，巩固绿化成果。

　　大力实施水利提升战略。按照优先利用地表水、严格控制地下水、高效配置引黄水、科学利用污废水、大力推行节约用水的思路，加强水生态建设，努力破解“水瓶颈”制约。今年重点抓好水系建设十大重点工程，即神头泉水源保护与生态修复工程、七里河市区段河道整治与生态修复工程、塞上灌区改扩建工程、恢河综合整治六期工程、山阴县桑干河湿地公园、怀仁县鹅毛河湿地公园、怀仁县金沙滩湿地公园、应县塔北水景公园、平鲁大梁湿地公园、右玉县海子湾水库下游河道治理工程。

　　大力推进节能减排和矿山生态治理修复。加大淘汰落后产能力度，积极实施节能工程和污染治理工程，从源头上治理生态环境污染。突出抓好大气污染防治，开展城市燃煤烟尘、扬尘整治。加强重点企业监管，强化电厂燃煤机组脱硫脱硝。加快推进城市集中供热，尽快彻底取缔燃煤小锅炉，推动城中村居民使用清洁能源。加大建筑工地、工业企业及车辆运输环节等扬尘治理力度。多措并举，加快改善城市空气质量。全面推进采矿沉陷区、采空区、水土流失区、煤矸石山、粉煤灰库生态修复工程，促进生态恢复，防止地质灾害发生。

　　推进生态文明制度建设。继续坚持“一矿一企治理一山一沟”、“挖一吨煤栽一棵树”的好做法，加强煤炭可持续发展基金管理使用，严格执行矿山环境恢复治理保证金制度，健全资源有偿使用制度和生态补偿制度，严格规划环评和项目环评，形成有利于环境保护的长效机制，促进绿色发展。

　　各位代表，生态建设已经成为朔州的一大品牌。我们一定要珍惜荣誉，再接再厉，继续大力弘扬右玉精神，不断谱写生态建设新篇章，努力建设塞外绿洲、塞上明珠!

　　(六)大力加强文化建设，加快建设文化强市

　　引深精神文明创建活动。传承优秀传统文化，大力弘扬右玉精神，加强社会主义核心价值体系建设。推进公民道德建设工程，加强社会公德、职业道德、家庭美德和个人品德教育，提升市民素质。深化群众性精神文明创建活动，培育良好社会风尚，提高城市文明水平。

　　着力构建公共文化服务体系。政府要履行好发展公益性文化事业的责任，大力实施文化惠民工程。加强文化遗产保护，开展文化遗产和文化资源数字化建设。加强基层文化体育活动场所建设，完善新闻大楼、博物馆、图书馆等重点文化工程，建设市级数字多厅影院。继续实施好农村电影放映和送戏下乡等工程，丰富群众精神文化生活。广泛开展全民健身运动，促进群众体育和竞技体育全面发展。

　　加快发展文化产业。深化文化体制改革，推动文化事业全面繁荣、文化产业快速发展。设立文化产业发展专项资金，加强与中国文化传媒集团等著名文化企业合作，支持文化精品创作，促进文化创意、演艺娱乐、文化旅游等产业加快发展。精心打造朔州边塞文化旅游节，塑造特色文化品牌，提升朔州形象。

　　文化是人民的精神家园。朔州自古为战略要地，在中华文明发展史上具有重要影响。朔州是著名的革命老区，曾经为民族独立和人民解放付出巨大牺牲。朔州是邓小平同志亲自选定的改革开放试验田，是国家重要的能源工业基地，是右玉精神的发祥地，承载了改革开放的时代精神和艰苦奋斗的优良传统，为我国现代化建设做出重要贡献。我们要像开采煤炭资源一样挖掘文化资源，像抓经济建设一样抓文化建设，让朔州独特的文化资源大放光彩!

　　(七)加大民生改善和社会事业发展力度，努力让人民过上更好的生活

　　保障和改善民生是一切工作的出发点和落脚点。在继续做好去年兴办的各件实事的同时，我们将进一步加大投入力度，大力实施惠民工程，加快发展各项社会事业。

　　稳定和扩大就业。实施更加积极的就业政策，加强就业服务工作，推进劳动就业和社会保障公共服务信息化平台建设，做好以高校毕业生为重点的青年就业工作。深入开展创业型城市创建活动，落实好金融、税收等方面的就业扶持政策，以创业带动就业。全面推行劳动合同和备案制度，规范企业用工。加大农民工权益保护力度，保障农民工按时足额领到工资。

　　完善社会保障体系。城镇职工基本养老、医疗、失业、工伤、生育保险参保率全部达到95%以上。城乡居民基础养老金再提高10元，达到65元。企业退休人员基本养老金提高10%。城镇居民医保和新农合财政补助标准提高40元，达到每人每年280元。城乡低保保障标准每人每月分别提高30元、24元，达到338元、172元。扩大非公有制经济单位职工参加社会保险覆盖面，加快实现社会保障全民覆盖目标。积极发展社会福利和慈善事业，加强社会养老服务体系建设，做好残疾人保障工作，完善社会救助体系。

　　继续推进保障性住房建设。新建各类保障性住房11490套，完成8700户农村危房改造任务。建立公平的保障性住房分配管理制度，切实保障低收入困难家庭的基本住房需求。落实房地产市场调控政策，促进房地产市场健康发展。

　　全面发展各级各类教育。加强公立幼儿园建设，推动学前教育加快发展。全面推进中小学标准化建设，促进义务教育均衡发展。加强市区学校建设，加快解决班容量大的问题。持续推进高中教育内涵特色发展，进一步提升高中办学水平。大力发展职业教育。职业教育学校要围绕地方产业发展，加强与省内外优质职业教育资源合作办学，扩大办学规模，提高服务经济发展的能力。进一步发展高等教育。朔州电力学院要完善一期工程，扩大招生规模，积极推进二期工程。朔州师专要搞好独立招生办学。及早开工建设怀仁陶瓷职业技术学院。全面推进素质教育，加强教师队伍建设，着力提高教育质量。

　　加快发展医疗卫生事业。深化医药卫生体制改革，在各级医疗单位全面实施基本药物制度，加快公立医院改革。积极推进乡镇卫生院标准化建设，加强乡村医生队伍建设，选聘医学院校毕业生充实乡村医院。全面开展国家级计划生育优质服务先进县创建活动，推进免费孕前优生健康检查项目全覆盖，提升计划生育工作水平，提高人口素质。

　　(八)深入推进转型综改试验区建设，开创改革开放新局面

　　按照转型综改试验区实施方案，围绕产业转型、生态修复、城乡统筹、民生改善四大任务，加快建立适应科学发展的体制机制，重点在先行先试和改革创新上取得突破。积极创新用地保障机制。认真落实城乡建设用地增减挂钩等试点政策，加大土地复垦和盐碱地开发利用力度，保障用地需求。强化建设用地考核，提高供地门槛，促进土地节约集约利用。加快完善金融服务体系。发展资本市场，支持企业上市和发行债券。搭建银企合作平台，健全融资担保体系，扩大信贷规模。规范壮大民营金融机构，提升金融体系组织机构建设水平，不断提高金融机构服务地方经济的能力。积极创新产业转型促进机制。发挥政府主导作用，建立健全新兴产业发展援助机制。发挥市场机制作用，坚持以资源换项目、换资金、换技术，大力推行“一矿一企”等好做法，积极建立资源型产业与非资源型产业均衡发展机制。“一矿一企”，既是煤炭企业回报家乡、回报社会、担当社会责任的理性选择，也是煤炭企业实现以煤为基、多元发展、增强抵御市场风险能力的正确发展方向。煤炭企业要抓住机遇，乘势而上，与朔州同转型、共跨越。

　　进一步加大开放力度。加强与京津和周边地区交流合作，积极承接长三角、珠三角地区产业转移。创新招商引资方式，推动引资、引技、引智有机结合，提高招商引资的综合优势和总体效益。深化与驻朔中央企业、省属企业的合作，加强与高校、科研院所以及省直厅局的合作，落实好各项战略合作协议。充分发挥中煤平朔、中煤金海洋、中电国际、山西煤销集团、同煤集团等大企业以及开发区、八个工业园区在招商引资中的平台和载体作用，促进项目和各类生产要素向园区和重点企业集聚。

　　各位代表，朔州是伴随着改革开放成长起来的一座年轻城市。改革、开放、创新，是朔州发展的不竭动力。转型综改试验区建设为朔州转型跨越发展提供了新的难得机遇，早转型早主动，我们一定要抢抓机遇，先行先试，勇于创新，在全省率先走出资源型地区转型跨越发展的新路子!

　　(九)坚持不懈抓好安全生产和社会稳定工作，为经济社会发展创造和谐环境

　　进一步强化安全生产理念。始终坚持把安全生产作为头等大事，时刻牢记安全生产是第一底线，牢固树立“抓经济发展是政绩，抓安全生产也是政绩”的理念，怀着敬畏生命、敬畏责任、敬畏制度之心，毫不放松地抓好安全生产工作。

　　进一步强化两个主体责任。全面贯彻安全第一、预防为主、综合治理的方针，进一步强化政府的安全监管责任，严格落实企业的安全生产主体责任。各级安全生产监管部门要对各行业的安全生产实行综合管理，依法开展监督检查。各行业主管部门要加强行业安全管理，强化日常监管。各类企业必须严格执行安全生产法律法规，加大安全生产投入，推进安全生产标准化建设，强化安全生产基础。深入开展以煤矿为重点覆盖各行业各领域的安全隐患排查治理，扎实推进“打非治违”行动，严厉打击私挖滥采行为。强化食品药品安全监管，提升食品药品安全保障水平。严肃对待事故，严格责任追究，严格落实安全生产目标责任考核“一票否决制”。坚决遏制重特大事故，减少一般性事故，促进安全生产形势稳定好转、持续好转、根本好转。

　　加强和创新社会管理。建立健全重大决策社会稳定风险评估机制，完善信访工作和矛盾调处机制，切实解决好土地征用、房屋拆迁、劳资纠纷等事关人民群众切身利益的问题。加强城市社区建设，健全基层社会管理和服务体系。深化“平安朔州”建设，完善社会治安防控体系，严厉打击各类违法犯罪行为，提高人民群众安全感。健全应急体系，有效应对和处置各类突发事件。扎实做好第三次全国经济普查。支持国防后备力量建设，做好双拥、优抚安置和人防工作。加强社会舆论引导，促进社会和谐。

　　(十)加强政府自身建设，优化发展环境

　　按照科学执政、民主执政、依法执政的要求，努力建设法治政府、服务政府、责任政府、廉洁政府、创新政府，为转型跨越发展创造良好环境。

　　严格依法行政，提高民主法治水平。认真执行人大及其常委会的决议决定，自觉接受监督。支持人民政协履行职能，健全协商民主制度。支持人民法院、检察院依法独立公正行使职权。加强政府法制建设，规范政府行为，严格按照法定权限和程序行使权力、履行职责，善于运用法治思维和法治方式，深化改革，推动发展，化解矛盾，维护稳定，促进社会公平正义。

　　转变政府职能，创优政务服务环境。认真贯彻国务院关于机构改革和职能转变的精神，积极转变职能，切实提高政府管理科学化水平。深入推进行政审批制度改革，继续简政放权，精简审批事项，规范行政许可。发挥好政务服务中心作用，管理运行好行政审批绿色通道和公共资源交易平台，为群众提供更加高效便捷的服务。

　　改进工作作风，加强干部队伍建设。严格执行中央关于改进工作作风、密切联系群众的各项规定，精简会议文件，改进会风文风，克服形式主义、官僚主义。加强公务员管理和培训，不断提高干部素质。各级领导干部要深入基层调查研究，向群众学习，拜人民为师，夙夜在公，恪尽职守，求真务实，真抓实干，创造性开展工作，做出实实在在的工作业绩。市直部门要引深“六个一”活动，以实际行动推动转型跨越发展。

　　坚持为民务实清廉，加强廉政建设。认真落实政府系统党风廉政建设责任制，完善惩治预防腐败体系，让权力在阳光下运行。加强行政监察和审计监督，严肃查处各类违法违纪案件。大力弘扬右玉精神，坚持勤俭办一切事情，厉行勤俭节约，反对铺张浪费，压缩一般性开支，把有限的资金用在人民群众最需要的地方。

　　各位代表，转型跨越发展任重道远，全面建成小康社会前景灿烂。让我们紧密团结在以习近平同志为核心的党中央周围，深入贯彻落实党的十八大精神，解放思想，开拓创新，凝聚力量，攻坚克难，切实抓好今年我市经济社会发展各项目标任务的落实，不断开创朔州转型跨越发展的新局面，努力创造朔州人民更加美好的新生活!
